# The prevalence of human papillomavirus in oropharyngeal cancer in a New Zealand population

**DOI:** 10.1371/journal.pone.0186424

**Published:** 2017-10-19

**Authors:** Rebecca Lucas-Roxburgh, Jackie Benschop, Bruce Lockett, Ursula van den Heever, Ruth Williams, Laryssa Howe

**Affiliations:** 1 Institute of Veterinary, Animal, and Biomedical Sciences, Massey University, Palmerston North, New Zealand; 2 Molecular Epidemiology and Public Health Laboratory, Hopkirk Research Institute, Massey University, Palmerston North, New Zealand; 3 Histopathology Department, MedLab Central Ltd, Palmerston North, New Zealand; Fondazione IRCCS Istituto Nazionale dei Tumori, ITALY

## Abstract

**Background:**

The incidence of oropharyngeal cancer (OPC) in New Zealand (NZ) has more than doubled over the last 14 years with 126 cases in 2010. Overseas studies have shown that human papillomavirus (HPV) plays a significant role in the development of these cancers. However, the role of HPV in OPC and the burden on the NZ health system is unclear.

**Aim:**

The aim of the study was to determine the prevalence and the genotypes of HPV associated with OPC in New Zealand.

**Methods:**

In this study, 621 OPC were identified from cancer registry data from 1996–98, 2003–05, and 2010–12. Biopsies of 267 cases were then retrieved from laboratories throughout New Zealand. p16 immunohistochemistry and a human beta globin PCR were performed on all specimens. HPV genotyping was performed on all beta globin positive specimens using real-time PCR with melt analysis.

**Results:**

Using a p16/PCR algorithm, 77.9% (95% CI: 71.1–83.5%) of cases were attributable to HPV. Of these, 98.5% were HPV 16 positive. There was also one case each of HPV 33 and 35. The percentage of HPV positive cases increased from 61.9% (95% CI: 40.9%– 79.2%) in 1996–98 to 87.5% (95% CI: 79.8%– 92.5%) in 2010–12. Results from the multivariable model, adjusted for sex and ethnicity found statistically significant associations between HPV positivity and timeframe (OR: 5.65, 95% CI: 2.60–12.30, 2010–12 vs 1996–98), and between HPV positivity and patient age (OR: 0.55, 95% CI: 0.33–0.99, ≥61 years vs ≤60 years).

**Conclusions:**

This data is consistent with data from other developed countries showing an increase in cases of HPV positive OPC in New Zealand, and the majority of cases being attributable to HPV 16. These results support the recent inclusion of males into the nationally funded immunization schedule for Gardasil^®^ 9.

## Introduction

Head and neck squamous cell carcinomas (HNSCC) comprise cancers of the oral cavity, larynx, hypopharynx, and oropharynx [1]. The incidence of HNSCC in developed countries has decreased in parallel with a decrease in the number of individuals that smoke [2,3]. However, the incidence of oropharyngeal cancers (OPC) has increased [4]. The proportion of cases reported to be due to human papillomavirus (HPV) varies considerably between studies and the variation is dependent on the population studied, location of the tumour, and method of HPV detection [5]. HPV 16 is a recognised carcinogen in the oropharynx, while there is limited evidence for the role of HPV 18 in OPC, and insufficient evidence for other HPV types [6]. Worldwide over 90% of HPV-positive OPC are due to high risk HPV 16 [7], with as many as 97% of cases reported by some studies to be attributable to HPV 16 [8,9]. Other high risk HPV types such as types 18, 31, and 33 have also been detected [7,9,10].

In the past, the majority of HNSCC was seen in the larynx and oral cavity and was associated with smoking and heavy alcohol consumption [4]. The increasing incidence of HPV-positive cancer of the oropharynx (including tonsillar and base of tongue cancers) over the last three decades is thought to be due to changing sexual behaviours and this is represented in the risk factors for the disease [4,11,12]. Data from the US, Australia, and Sweden has shown an increase in risk factors such as the occurrence of premarital sex, oral sex, the number of lifetime partners, and a reduction in the age of sexual debut in recent birth cohorts [13]. Other risk factors include marijuana use and diet and nutrition [14,15].

The number of cases of OPC caused by HPV in New Zealand is unknown, however, when looking at anatomical site alone there has been an approximately threefold increase in the number of OPC cases from 35 cases in 1996 to 126 cases in 2010 (Ministry of Health, 2014). In contrast, the incidence of oral cavity cancers has remained steady during the same time period [16]. Currently there is no published data on the contributions of various HPV types to OPC in New Zealand. It is likely New Zealand’s cases follow international data with HPV 16 responsible for the vast majority of cases. Thus, this study aims to determine the prevalence and the genotypes of HPV associated with OPC in New Zealand.

## Materials and methods

Ethics approval was obtained from the Health and Disabilities Ethics Committee (reference: 14/STH/128). Participant privacy was preserved by de-identification of samples.

### Participant recruitment and sample collection

Cases were identified by a New Zealand cancer registry search of all cases of oropharyngeal cancer of ICD-10 codes C01, C05.1–5.2, C09.0–9.9, and C10.0–10.9 from 1996–98, 2003–05, and 2010–12. The New Zealand cancer registry is a population based register of all primary malignant diseases diagnosed in New Zealand. The information available from the registry comprises: patient demographics (age, sex, and ethnicity) and tumour specific information (tumour site, morphology, grade, extent, and diagnosis date, laboratory code and basis for diagnosis (histology, cytology etc)). Data was cross checked with National Health Index (NHI) data to identify patients that were deceased. Study information sheets and consent forms were sent to all living patients whose samples were stored in laboratories with five or greater specimens in storage as per the laboratory code from the cancer registry data, and with current and complete address details. In addition the whānau (extended family) of deceased participants identified as Māori from the Auckland region were requested to give consent for these specimens to be used, as per local Iwi (tribe) wishes. All participants provided written informed consent prior to specimens being requested from storage. After specimen requests were made, laboratories sent the specimens (n = 267), a copy of the pathology report, and any existing p16 slides (n = 57) to Massey University.

### p16 IHC

Samples with a historic p16 slide had the existing slide used in this study. Samples were recoded and read blinded. All other samples had p16 IHC performed at a diagnostic medical laboratory (MedLab Central Ltd, Palmerston North, New Zealand). Formalin fixed paraffin embedded (FFPE) tissue was used for IHC. Sections were cut at four microns and baked onto Superfrost Plus slides at 60°C for 60 minutes. Staining was performed on the Ventana Benchmark Ultra (Ventana Medical Systems, Tucson, USA), with antigen retrieval performed on board, using Ventana CC1 buffer for 32 minutes at 100°C. A 1:150 dilution of the p16 (INK4a) antibody (G175-405, product code 551153) (BD Biosciences, North Ryde, Australia), was incubated on the slide for eight minutes at 36°C. The detection of p16 was then visualised using the Ventana Optiview DAB kit (Ventana Medical Systems). A multi-tumour block positive control containing a serous ovarian carcinoma was included with each run of samples. All slides were read independently by two pathologists (BL and UV), and discordant cases reviewed to reach a consensus. Samples showing strong diffuse staining in the nucleus and cytoplasm of greater than 75% of tumour cells were considered positive. Samples showing staining in 10% to 75% of tumour cells were considered focally positive, and samples with staining in less than 10% of tumour cells were considered negative.

### DNA extraction

A 10 μm slice of representative FFPE tumour block from each case with sufficient material (n = 224) was cut for DNA extraction using a new blade between each sample to avoid cross contamination of samples. DNA extractions were performed using the DNeasy^®^ Blood and Tissue kit (Qiagen, Hilden, Germany) as per manufacturer’s instructions for tissue. A control extraction was included with each set of samples. The blank extractions were then used as negative controls in the beta globin and HPV 16 qPCRs. A pre-treatment for paraffin embedded samples was included, and a final elution volume of 100 μL was used. The quality of extracted DNA was assessed using a Nanodrop^™^ Spectrophotometer (Thermo Fisher Scientific, Waltham, USA).

### Beta-globin qPCR

The presence of amplifiable DNA was assessed on all cases with extracted DNA by qPCR targeting the human beta-globin gene using the PC03 and PC04 primers [17]. All primers used are shown in Table 1. Each 20μL reaction mix contained: 0.25 μM each primer, and 1X Fast Start SYBR Green Master (Roche Diagnostics, Basel, Switzerland). Up to 50 ng of extracted DNA was used as template. Cycling conditions were 95°C for 10 minutes, followed by 40 cycles of: 95°C for 15 seconds, 55°C for 30 seconds and 72°C for 30 seconds. PCR was followed by a melt curve from 75–85°C with 0.2°C increments and a three second hold. A sample was considered positive if it produced a *T*_m_ of 81.0°C (±1.0°C). Samples showing low amplification on the qPCR were visualised using gel electrophoresis through a 1.0% agarose gel containing 1X SYBR^®^ Safe (Thermo Fisher Scientific) to confirm product amplification.

**Table 1:** Primer sequences and PCR conditions.

Primer name	Primer sequence (5’-3’)	Annealing temp	Gene target/ position	Product Size (bp)	Reference
16F cloning	GAT CAG TTT CCT TTA GGT CG	62°C	HPV 16^1^	1775	Primers designed for this project
16R cloning	GGT ACC TGC AGG ATC AGC CAT		(7014-885bp)		
PC03	ACA CAA CTG TGT TCA CTA GC	55°C	Human beta globin gene^2^	110	Saiki et al [17]
PC04	CAA CTT CAT CCA CGT TCA CC		(827-937bp)		
GP5+	TTT GTT ACT GTG GTA GAT ACT AC	49°C	HPV L1 gene^1^	140	de Roda Husman et al [18]
GP6+	GAA AAA TAA ACT GTA AAT CAT ATT C		(6624-6765bp)		
16F	GTG GAC CGG TCG ATG TAT GTC T	62°C	HPV 16 E6^1^	209	Dictor and Warrenhalt [19]
16R	TCC GGT TCT GCT TGT CCA GC		(496-704bp)		

^1^ Genbank accession number: KO2718

^2^ Genbank accession number: L26478

### HPV 16 qPCR

The presence of HPV 16 was assessed on all cases with a positive beta globin qPCR result. A qPCR with melt curve analysis was developed using previously described primers [19]. Each reaction mix contained: 0.25 μM each primer, and 1X Fast Start SYBR Green Master (Roche Diagnostics). Up to 50 ng of extracted DNA was used as template. Cycling conditions were 95°C for 10 minutes, followed by 40 cycles of: 95°C for 15 seconds, 62°C for 30 seconds and 72°C for 30 seconds. PCR was followed by a melt curve from 70–80°C with 0.2°C increments and a five second hold. A sample was considered positive if it produced a *T*_m_ of 77.6°C (±1.0°C).

A positive control was made from cloned DNA of a HPV 16 positive clinical sample provided by MedLab Central Ltd (Palmerston North, New Zealand). Cloning was performed using a pGEM^®^-T Easy kit with JM109 High Efficiency Competent Cells (Promega, Madison, USA), according to the manufacturer's instructions with blue/white selection. The cloned plasmid contained an approximately 1.8 kb fragment of the HPV 16 genome that included the E6 and E7 gene sequence amplified using the primers detailed in Table 1. Before being used as a positive control, the cloned plasmid was subjected to automatic dye-terminator cycle sequencing with BigDyeTM Terminator Version 3.1 Ready Reaction Cycle Sequencing kit and the ABI3730 Genetic Analyzer (Applied Biosystems, Life Technologies Corporation, Carlsbad, California, USA) to confirm genomic sequence using both the forward and reverse primers. The sequences obtained were compared using the NCBI Blast database to other published sequences available from GenBank [20].

Duplicate standard curves from eight, 10-fold serial dilutions starting from 9.56 x 10^7^ copies of HPV 16 were used to determine the limit of detection of the HPV 16 qPCR.

### Confirmation of non-HPV 16 types

Samples that were p16 positive / focally positive, and HPV 16 negative were subject to qPCR using the GP5+/6+ primers [18]. Each reaction contained 0.2 μM each primer, 2.0 mM MgCl_2_, 1X PCR buffer, 0.3 mM each dNTP, 1 unit Platinum^™^ *Taq* (Invitrogen, Carlsbad, USA), 1.5 μM Syto^®^ 9 (Invitrogen). Up to 50 ng extracted DNA was used as template. Cycling conditions were 95°C for 10 minutes, followed by 40 cycles of: 95°C for 15 seconds, 49°C for 30 seconds and 72°C for 30 seconds. PCR was followed by a melt curve from 70–85°C with 0.2°C increments and a five second hold. Known HPV 16, 18, 33, and 52 positive samples were included in each run. Samples with a *T*_m_ in the range of 76.0–81.0°C were considered positive for HPV DNA. The GP5+/6+ PCR could not reliably discriminate HPV genotypes therefore, the product of samples positive for the GP5+/6+ PCR were purified using the PureLink^®^ PCR purification kit (Invitrogen, Carlsbad, CA, USA). The HPV type present was then confirmed by sequencing as previously described.

### Data handling and statistical analysis

The continuous variable age was categorised into a binary variable: aged 60 years or younger, and 61 years or older. Ethnicity was classified into NZ European, NZ Māori, and other.

To investigate the association between HPV positive OPC and putative risk or confounding factors (age, sex, ethnicity, and time period), each factor was tested individually for significance at p < 0.2 in a logistic regression model using the software package R version 3.2.0 (R Development Core team, 2010, R Foundation for Statistical Computing, Vienna, Austria). A multivariable model was built by a stepwise selection process, retaining all variables significant at p < 0.05 and any confounding variables. Once a main effects model was built two-way interaction terms were introduced to the model and retained if significant at p < 0.05.

## Results

### Study population

The national dataset from the cancer registry comprised a total of 621 cases from the 1996–98 (n = 113), 2003–05 (n = 185), and 2010–12 (n = 323) time periods. Diagnosis was based on histology in 94.8% (n = 589) of cases, cytology in 4.6% (n = 29) of cases and other non-specified tests in 0.6% (n = 3) of cases. The proportion of cases diagnosed on histology versus cytology was consistent over all time frames. The mean age of an OPC patient was 59.2 years. NZ Europeans made up 68.8% of the national cases, and NZ Māori a further 10.5%. There was an approximate 4:1 ratio of males to females.

Of the 344 living patients identified from the national dataset, 303 were able to be contacted, of which 52% (157/303) gave consent. Of the 157 cases, 109 specimens could be retrieved. There were 277 deceased patients identified in the national dataset. Of the 277 cases, 158 specimens were retrieved for use in the study.

Thus, the total study population comprised 267 cases with retrievable specimens, and represented all major centres. Between four and 61 cases were retrieved from each of the 15 laboratories involved. The demographic factors of the national dataset and study population were comparable as shown by the high p-values for age, sex, ethnicity and timeframe (Table 2). Therefore, the study population was considered to be reflective of the national dataset.

**Table 2:** Comparison of demographic factors of the study population and national data.

		National dataset (n = 621)	Excluded cases (n = 354)	Study population (n = 267)	p-value^1^
Mean age at diagnosis		59.2 years	58.6 years	60.1 years	0.30
Sex:	Male	492 (79.2%)	283 (79.9%)	209 (78.3%)	0.82
	Female	129 (20.8%)	71 (20.1%)	58 (21.7%)	
Ethnicity:	NZ European	427 (68.8%)	237 (66.9%)	190 (71.2%)	0.77
	NZ Māori	65 (10.5%)	36 (10.2%)	29 (10.9%)	
	Other	118 (19.0%)	73 (20.6%)	45 (16.9%)	
	Not stated	11 (1.7%)	8 (2.3%)	3 (1.0%)	
Timeframe:	1996–98	113 (18.2%)	62 (17.5%)	51 (19.1%)	0.83
	2003–05	185 (29.8%)	102 (28.8%)	83 (31.1%)	
	2010–12	323 (52.0%)	190 (53.7%)	133 (49.8%)	
Deceased		277 (44.6%)	119 (33.6%)	158 (59.2%)	<0.01
Living		344 (55.4%)	235 (66.4%)	109 (40.8%)	

^1^ p-value calculated compares the study population to the national dataset.

### p16 IHC

**Fig 1:**
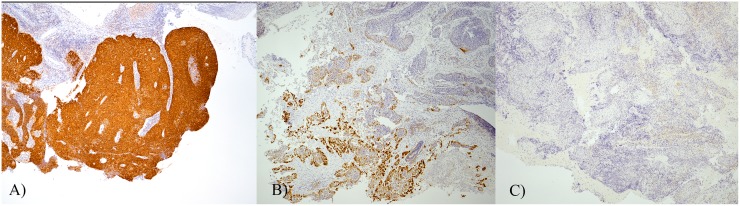
p16 staining patterns. A) Positive sample with >75% of tumour cells showing strong diffuse staining (magnification 40 x) B) Focally positive samples with 10–75% of tumour cells stained (magnification 40 x), and C) a negative sample with <10% of tumour cells stained (magnification 40 x).

p16 results were categorised as positive, focally positive, or negative (Fig 1). Consensus was achieved in all cases. A positive result was seen in 58.4% (156/267) of cases, 7.9% (21/267) were focally positive, and 27.3% (73/267) were negative. The remaining 6.4% (17/267) of cases had no tumour present in the block (n = 10), or had insufficient material present (n = 7).

There were 57 cases with a historic p16 slide. When comparing study p16 results to original results from cases with historic p16 slides, concordance was seen in 49/49 positives, 1/2 focally positives, and 5/5 negatives. The discordant focally positive case was identified as negative by our study. The remaining case had a slide sent for the study but no p16 result in the original pathology report.

### Beta-globin, HPV 16, and GP5+/6+ qPCR

**Fig 2:**
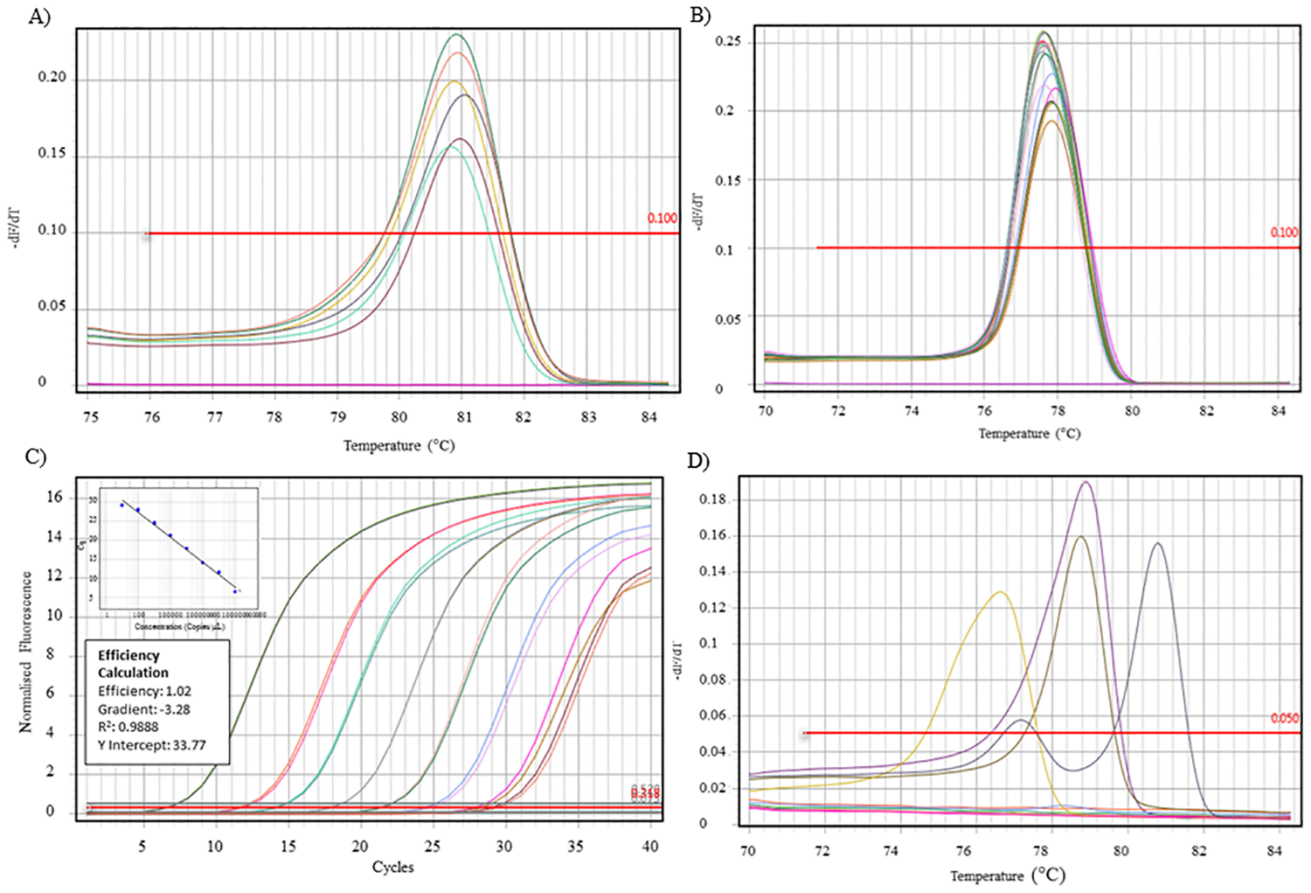
qPCR figure panel. A) Melt curve of beta-globin qPCR. Positive samples shown with *T*_m_ of 81.0°C (±1.0°C). B) Melt curve of HPV 16 qPCR. Positive samples shown with *T*_m_ of 77.6°C (±1.0°C). C) Standard curve of HPV 16 clone control serial dilutions. D) Melt curve of GP5+/6+ qPCR. Positive samples shown with *T*_m_ in the range of 76.0–81.0°C.

DNA could be extracted from 224 samples. Following the beta-globin qPCR (Fig 2) a further 4.0% (9/224) were excluded due to a lack of amplifiable DNA. HPV16 qPCR was performed on all beta globin positive samples. Serial dilutions of a HPV 16 clone control showed the assay was capable of detecting 26 copies of the HPV 16 E6 target sequence (Fig 2). HPV 16 was detected in 74.4% (160/215) of cases.

Results of the GP5+/6+ qPCR (n = 17) were 82% (14/17) negative, and 18% (3/17) positive (Fig 2). Of the three positive samples, HPV was confirmed by sequencing in two cases, one case each of HPV 33 and 35. The HPV type could not be detected in the remaining case. Overall, high risk HPV was detected in 75.3% (162/215) of cases. The detection of a high risk HPV type by PCR/sequencing and the p16 result is shown in Table 3.

**Table 3:** p16 result, and detection of a high risk HPV type by PCR/sequencing in archived oropharyngeal cancer biopsy samples.

		High risk HPV		Totals
		Positive	Negative	
p16 result	Positive	134	6	140
	Focally positive	11	9	20
	Negative	17	37	54
Totals		162	52	214

For the purpose of further analysis, a sample is considered positive if positive by p16, and positive for either the HPV 16 qPCR, or a high risk HPV type was confirmed by sequencing. A negative sample is negative by p16, and HPV 16 qPCR negative. This approach resulted in an overall study prevalence of HPV positive OPC of 77.9% (95% CI: 71.1–83.5%). HPV 16 accounted for 98.5% of HPV positive OPC.

### Statistical analysis

The proportion of cases attributable to HPV increased from 61.9% (95% CI: 40.9%– 79.2%) in 1996–98 to 63.8% (95% CI: 49.5%– 76.0%) in 2003–05, and finally to 87.5% (95% CI: 79.8%– 92.5%) in 2010–12.

The mean age of an HPV negative patient was 66.2 years (95% CI: 65.8–66.4), compared to 56.8 years (95% CI: 56.6–56.9) for an HPV positive patient. The univariate analysis of putative risk factors showed age (p < 0.01) and timeframe (p < 0.01) to be significant (Table 4). In the final multivariable model (Table 4) there was a statistically significant association between HPV positivity and time frame (OR: 5.65, 95% CI: 2.60–12.30, 2010–12 vs 1996–98). A statistically significant association between HPV positivity and patient age was also found (OR: 0.55, 95% CI: 0.33–0.99, ≥61 years vs ≤60 years). These estimates were adjusted for sex and ethnicity.

**Table 4:** Univariable and multivariable analysis of putative risk factors associated with having an HPV positive tumour.

Variable		Univariable analysis			Multivariable analysis		
		Odds Ratio	95% CI	P value	Odds Ratio	95% CI	P value
Age	≤60	REF^1^			REF^1^		
	≥61	0.48	0.28–0.82	<0.01	0.55	0.33–0.99	0.05
Sex	Female	REF^1^			REF^1^		
	Male	1.50	0.79–2.78	0.21	1.36	0.68–2.66	0.38
Ethnicity	NZ European	REF^1^			REF^1^		
	NZ Māori	1.82	0.74–5.15	0.22	1.49	0.55–4.52	0.45
	Other	1.07	0.52–2.27	0.86	1.14	0.53–2.53	0.74
Timeframe	1996–98	REF^1^			REF^1^		
	2003–05	1.75	0.84–3.67	0.14	1.76	0.83–3.81	0.14
	2010–12	5.90	2.82–12.62	<0.01	5.65	2.60–12.30	<0.01

^1^ REF is the baseline level (OR = 1.00)

## Discussion

The increase in the proportion of HPV positive cases between 1996–98 and 2010–12 seen in this study augments Ministry of Health data showing over a three-fold increase in OPC case numbers, regardless of HPV status between 1996 and 2010. Overall, the results of this study showed that the majority of OPC cases in New Zealand are caused by HPV. An HPV positive patient is more likely to be under 60. The younger age for HPV positive patients and increase in prevalence from this study are consistent with international data [7,12,21]. Other developed counties have seen increases in the proportion of HPV positive cases ranging from 25% in Sweden between 1970 and 2007 [22], to 225% in the United States between 1988 and 2004 [23]. Moreover, there were no significant differences between the national dataset and study population in terms of patient age, sex, ethnicity, or timeframe of diagnosis, showing this study’s results can be extrapolated to the wider New Zealand setting.

There was a higher proportion of deceased patients in our population than the national dataset (59% versus 45%) and this is likely due to the different consenting requirements. Due to the poorer prognosis of HPV negative tumours [13], the inclusion of a higher proportion of deceased cases may have caused an over-representation of HPV negative cases. A small regional New Zealand study performed as retrospective audit, which did not require any patient consent, showed an overall prevalence of HPV positive OPC of 63% based on p16 results alone [24]. Our overall prevalence of HPV positive cases using an accepted p16/PCR algorithm [21] was 77.9%. It is therefore unlikely that HPV negative cases were overrepresented in our study.

We requested 389 specimens from laboratories throughout New Zealand to capture our final study population of 267 cases. There were several reasons why cases were not retrieved, such as 1) there were logistical difficulties identifying cases (due to the later introduction of computerised records in some centres, and laboratory take-overs / mergers), 2) cases were diagnosed on cytology only and had no biopsy / cell block, 3), specimens had been destroyed in the 2011 Christchurch Earthquake, and 4) specimens had been removed from storage and could not be located. Given the many reasons for samples not being included and the final comparisons of our study population to the national dataset, there was no reason to suspect any systematic bias in our sample.

In New Zealand, laboratories are required by law to report any new diagnosis of cancer to the cancer registry. This generates complete national incidence data. Our study compared only risk factors with data available from the cancer registry (age, sex, ethnicity, and timeframe of diagnosis). It is important to note these are not the only risk factors for HPV positive OPC and other significant factors such as alcohol consumption, tobacco smoking and sexual behaviours [4,11,12] could not be assessed. We therefore cannot rule out possible differences between study participants and those excluded from the study.

It should be noted, that our final criteria for a positive result was both p16 positive and either HPV 16 qPCR positive, or a high risk HPV type confirmed by sequencing. These criteria resulted in 172 results from 267 cases for analysis. This approach excluded focally positive cases and those cases that were p16 negative but HPV 16 positive by PCR. The exclusion of focally positive cases, and use of the p16 positive cut-off at 75% of tumour cells stained, aligned our definition of a p16 positive result with that used in many other studies [8–10,12,21,25].

Although p16 is routinely used alone in the diagnostic setting [26], the use of an algorithm incorporating p16 and another method (PCR or ISH) is preferred in research [8,9,21,27]. Whilst some studies only perform PCR testing on p16 positive cases [21], a more common approach is for all samples to undergo PCR testing for HPV DNA [8–10]. This approach generates four possible result categories p16 positive/HPV positive, p16 negative/HPV negative, and the equivocal results of p16 positive/ HPV negative and p16 negative/ HPV positive [28]. Discrepancies between p16 and PCR results have been reported to be between 6% and 31% [8,29]. In this study, discordant p16/PCR results were seen in 12.8% (25/195) of our cases where, eight cases were p16 positive/ high risk HPV negative, and 17 cases were p16 negative/ HPV16 positive. In the p16 positive / HPV negative cases, it is possible that p16 is upregulated by non-HPV related mechanisms [30].

The viral load in tonsillar cancer has been reported to be between 1.54 x 10^2^ and 1.34 x 10^7^ copies per 50 ng of DNA [31]. Our HPV 16 qPCR was capable of detecting 26 copies of HPV, therefore it is unlikely that low levels of HPV are the reason for the eight p16 positive / high risk HPV negative results. Additionally, due to using a new blade for each sample section, and the inclusion of control DNA extractions in each run it is unlikely the p16 negative / HPV positive cases are due to contamination. These 17 cases may represent a transient /bystander HPV infection unrelated to the tumour [9,28]. Junor *et al* [29] reported that p16 negative, HPV DNA positive cases were a distinct clinical entity based on survival characteristics compared to p16 positive/HPV positive and p16 negative/HPV negative cases. Evans *et al* [8] also reported intermediate survival characteristics for patients with equivocal p16/HPV results. Our criteria for a negative p16 result was less than 10% of tumour cells stained. Interestingly 88% (15/17) of the HPV DNA positive /p16 negative samples showed patchy staining in less than 10% of tumour cells.

While multi-levelled result categories or scoring systems for p16 have been reported [32–34], more frequently cases with less than 70% of tumour cells stained are regarded as negative [8,10,21]. Our study had 7.9% (21/267) focally positive p16 cases which had variable staining patterns, and the percentage of cells stained ranged from 10–80%. The single case with 80% of cells stained was defined as focally positive as staining was only present in the cytoplasm. This is consistent with two previous studies by Chen *et al* [33] and Lewis *et al* [34] who reported 8.2% and approximately 4%, respectively, of OPC cases with partial p16 staining. The focal staining in both of these studies ranged from less than 5% of tumour cells to 90% of tumour cells, where the cases showing over 75% staining were considered focally positive based on additional criteria such as staining intensity [33,34]. Approximately half of our focally positive cases had HPV DNA detected by PCR. However, further work is required to determine if these cases contain transcriptionally active HPV and therefore are true HPV positive cases, or additional equivocal cases. Regardless, the differing published criteria and lack of international guidelines for p16 interpretation in OPC show clarity is needed. A simple cut off point may not be enough and other factors such as staining intensity, location, and confluence may need to be considered [33,34]. It is possible the age of the blocks contributed to the focally positive results in this study. When looking at each timeframe 5% from 2010–12 were focally positive, compared to 10% from 2003–05 and 15% from 1996–98. A reduced sensitivity and specificity of p16 on older specimens has been reported by Chenevert *et al* [12], however, their blocks were considerably older and dated back to 1956.

Worldwide HPV is responsible for over 90% of HPV positive OPC [7]. Co-infection is rarely described, and when present usually involves HPV 16 and another high risk type [9,10,35]. To date only HPV 16 is a recognised carcinogen in the oropharynx, while there is limited evidence for HPV 18 [6]. Co-infection was not assessed by this study. However, our findings of HPV 16 accounting for 98.5% of HPV positive cases, and the detection of HPV 33 and 35 is consistent with international data [35]. This study had one case in which the HPV type/s could not be determined. This case was p16 positive, HPV 16 negative and GP5+/6+ positive. The GP5+/6+ PCR is capable of detecting a broad spectrum of HPV types [18]. It is possible the sample contained a low risk HPV type not related to the cancer. The coincidental detection of low /intermediate risk types such as 6, 11, 32, 44, 53, 57 and 81 in OPC has been reported in a systematic review of HPV types in HNSCC by Kreimer *et al*, with HPV 6 the most commonly detected with a prevalence of 3.1% [35]. It is also possible this case contained a co-infection and therefore the types present could not be established by sequencing.

Based on our results, a HPV positive patient is more likely to be under 60, and diagnosed recently (2010–12). The prevalence of HPV positive OPC in females appears to be increasing at a faster rate than in males based on our study data. However, interpretation of this apparent trend is limited due to the low number of cases in females in this study. There were no HPV positive cases in females in 1996–98, although there was one case that was p16 positive that could not have DNA extracted. This is compared to 20% of p16/HPV DNA positive being in females in the 2010–12 time period. A statistically significant increase in New Zealand OPC cases in females of 2.1% per year between 1982 and 2010 has been previously reported [16]. Although Elwood *et al*, focused on tumour site alone without assessing HPV status, the results support the apparent increase seen in our study.

Chaturvedi *et al* [23] estimated that by 2020 the incidence of HPV positive OPC cases will outnumber the number of cervical cancer cases in the United States. Our data suggests the same scenario will likely be seen in New Zealand. The increasing prevalence of HPV positive OPC and the predominance in males supports the recent inclusion of males in the funded immunisation schedule for Gardasil ^®^9 in New Zealand. HPV types 16, 18, 6, 11, 31, 33, 45, 52, and 58 are included in Gardasil^®^9 [36]. Therefore, 99.3% of HPV positive cases with a detectable HPV type in this study contain types included in the vaccine. These results suggest that with adequate coverage, vaccination is likely to have a considerable impact on the future incidence of HPV positive OPC in New Zealand.

## Supporting information

S1 File Study database (participant demographics and lab results). (XLSX)
